# Women with internet related disorders- illustration of an in-depth clinical assessment and personnalized treatment approach

**DOI:** 10.1192/j.eurpsy.2024.92

**Published:** 2024-08-27

**Authors:** S. Achab

**Affiliations:** Psychiatry, Clinical and Sociological Research Unit, Faculty of Medicine Geneva University, Geneva, Switzerland

## Abstract

**Abstract:**

Towards a personalized response to public health issues of PIU in women, understanding female profiles of problematic internet users and their underlying psychosocial characteristics is a crucial preliminary step.

Mobile Problematic Internet Use (PIU) is most likely present in young females, with heavy pattern of use being specifically associated to some online activities including communication, buying, video gaming and video watching.

The present talk will introduce epidemiological data on female with PIU in Europe, and will afterwards describe clinical assessment and treatment of a young female suffering from mobile PIU.

Assessment found heavy social networks (SNs) use, being mainly explained by dysfunctional coping to low self-esteem and traumatic sexual experiences in her biography.

Psychotherapy consisted in CBT for excessive time devoted to SNs, relocating life-priorities and cognitive remediation targeting self-esteem, self-compassion, and assertiveness.

Behind quantitative data on PIU in female, each patient has specific needs for treatment that should be identified in other to address PIU.

Care process model used at ReConnecte since a decade allows for a comprehensive assessment of each PIU.

**Disclosure of Interest:**

None Declared

